# Comparative analyses of COVID-19 in-hospital mortality in people with HIV during SARS-CoV-2 pre-Delta, Delta, and Omicron waves

**DOI:** 10.1097/QAD.0000000000004323

**Published:** 2025-10-21

**Authors:** Seth Inzaule, Ronaldo Silva, Nathan Ford, Soe Soe Thwin, Jassat Waasila, Alimuddin Zumla, Meg Doherty, Janet Diaz, Silvia Bertagnolio

**Affiliations:** a Amsterdam Institute for Global Health and Development, and Department of Global Health, Amsterdam UMC, University of Amsterdam, Amsterdam, The Netherlands; b UNDP/UNFPA/UNICEF/WHO/World Bank Special Programme of Research, Development and Research Training in Human Reproduction (HRP), Department of Sexual and Reproductive Health and Research, World Health Organization; c Department of Global HIV, STI, Hepatitis Programmes, World Health Organization, Geneva, Switzerland; d National Institute for Communicable Diseases, Johannesburg, South Africa; e Division of Infection and Immunity, Center for Clinical Microbiology, University College London; f NIHR Biomedical Research Centre, UCL Hospitals NHS Foundation Trust, London, UK; g Department of AMR Surveillance, Prevention and Control World Health Organization, Geneva, Switzerland

**Keywords:** COVID-19, HIV, mortality, people with HIV, SARS-CoV2, vaccine

## Abstract

**Background:**

We investigated in-hospital mortality trends across waves of different SARS-CoV-2 variants and assessed the effect of COVID-19 immunization among people with HIV (PWH).

**Method:**

We analyzed individual-level data from the WHO Global Clinic Platform comprising 823 845 hospitalized children and adults from 61 countries. Survival analyses were used to assess the association of HIV co-infection with in-hospital mortality across SARS-CoV-2 pre-Delta, Delta, and Omicron variant waves.

**Findings:**

PWH experienced significantly higher in-hospital mortality compared to HIV-negative individuals across all variant waves. Adjusted hazard ratios (aHRs) for PWH mortality were 1.85 [95% confidence interval (CI) 1.76–1.93] during the pre-Delta wave, 1.58 (95% CI 1.42–1.74) during the Delta wave, and 3.07 (95% CI 2.75–3.42) during the Omicron wave. In-hospital mortality risk was notably higher in PWH with CD4^+^ cell count 200 cells/μl or less. While mortality declined modestly between pre-Delta and Delta waves in both HIV-negative (10% reduction) and HIV-positive populations (9% reduction), the decline was more substantial for HIV-negative individuals during the Omicron wave (from 18.9 to 8%) than for PWH (from 24.2 to 19.3%) relative to the Delta wave.

**Interpretation:**

Although in-hospital mortality among HIV-negative individuals declined markedly during the Omicron wave, reduction in PWH was less pronounced, leading to a relatively higher mortality risk for this group. These findings highlight the need for adherence to WHO recommendations on booster vaccinations and therapeutics for populations at elevated risk of severe COVID-19 outcomes, including PWH.

## Introduction

As of mid-July 2025, SARS-CoV-2, the virus responsible for COVID-19, has caused over 778.3 million cases and 7.09 million deaths, making it one of the deadliest pandemics of the 21st century [1]. The COVID-19 pandemic has largely been characterized by waves of infections, primarily driven by the emergence of variants of concerns (VoCs), some of which with increased transmissibility or immune escape capacity. These properties have enabled certain variants to rapidly dominate globally, despite the implementation of various infection control measures, including vaccination efforts. Although the pandemic is now largely under control, new infections persist, with approximately 1.01 million cases reported since the beginning of 2025, raising concerns about the emergence of new, highly transmissible variants, such as the circulating Omicron subvariant JN.1 [1,2].

Previous VoCs have exhibited distinct disease dynamics. Pre-Delta variants (Alpha, Beta, and Gamma) demonstrated relatively high transmissibility compared to the original SARS-CoV-2 strain. The Delta variant was associated with increased disease severity and mortality, whereas the Omicron variant displayed heightened transmissibility and immune evasion but generally reduced disease severity [2–5]. Despite these trends, understanding the differential impact of these variants is crucial, especially among vulnerable populations such as people with HIV (PWH).

While the overall risk of death declined during the Omicron variant wave, studies report that the relative decline may have been modest among certain groups previously identified as being at higher risk of COVID-19 mortality, such as individuals with compromised immunity [6,7].

PWH represent a key population with compromised immunity, predisposing them to opportunistic infections and a higher burden of noncommunicable diseases such as diabetes and hypertension – conditions that have been associated with severe or fatal COVID-19 outcomes [8–10]. PWH often face an elevated risk of severe infections because of their compromised immune systems, making them more susceptible to respiratory illnesses, including COVID-19. Previous studies have reported conflicting findings regarding the mortality risk associated with COVID-19 among PWH, with some indicating a higher risk of severe outcomes and others suggesting no significant difference compared to HIV-negative individuals [9,11–13]. This inconsistency underscores the need for further research into the interaction between HIV and COVID-19, particularly in the context of emerging variants[9,13]. Despite the substantial body of literature on COVID-19, there remains a critical gap in understanding how different SARS-CoV-2 variant waves affect the in-hospital mortality risk among PWH compared to HIV-negative populations. The evolving nature of the virus necessitates ongoing surveillance and research to inform public health strategies and clinical practices effectively. Certain VoCs may have emerged from individuals with compromised immunity, where prolonged long viral clearance and limited immune pressure allow the virus to adapt more effectively within the host [14–18]. For example, the mutation patterns seen in Omicron variant are similar to those found in viruses obtained from an immunocompromised individual with HIV before the widespread transmission of Omicron [18].

In this study, we aimed to assess the risk of in-hospital mortality among hospitalized COVID-19 patients, focusing on the effects of HIV status across different variant waves (pre-Delta, Delta, and Omicron) from hospitals in 61 countries. By analyzing the demographic and clinical characteristics of patients, as well as the impact of vaccination status and underlying comorbidities, we aimed to elucidate the mortality risks associated with COVID-19 among PWH especially as new variants emerge, and addressing the need for tailored strategies to protect and support this vulnerable group.

## Methods

### Data sources

We utilized anonymized individual-level data from the WHO Global Clinical Platform across 61 countries who contributed data of patients hospitalized with COVID-19 to the WHO Global Clinical Platform for COVID-19. The countries contributing data were: Australia, Bangladesh, Belarus, Belgium, Belize, Bolivia, Brazil, Burkina Faso, Cameroon, Canada, Chile, China, Colombia, Democratic Republic of Congo, Dominican Republic, Ecuador, Estonia, France, Gambia, Germany, Ghana, Greece, Guinea, Hungary, India, Indonesia, Iran, Ireland, Israel, Italy, Japan, Jordan, Kuwait, Malawi, Malaysia, Mexico, Netherlands, New Zealand, Niger, Nigeria, Norway, Pakistan, Panama, Peru, Philippines, Poland, Portugal, Republic of Korea, Romania, Russia, Singapore, South Africa, Spain, Sudan, Switzerland, Turkey, Ukraine, UK, USA, Zambia, and Zimbabwe. The design and implementation of this platform have been described previously in detail [13,19]. Data were contributed through a standardized case report form (CRF), available in both paper-based and electronic formats, as well as through local systems or databases. For locally entered data, relevant variables were mapped to the WHO CRF data dictionary and subsequently transferred to the WHO Clinical Platform hosted on REDCap.

The WHO CRF collects a standardized set of variables at hospital admission, daily during hospitalization, and at discharge. Collected information includes demographics, pregnancy status, country, vital signs, anthropometric measurements, underlying conditions, chronic medications, clinical features, immunization status, re-infection details, laboratory tests, therapeutics, admission to ICU, use of oxygen and mechanical ventilation, complications, and clinical outcomes (e.g. discharge, death, transfer to another facility, or remaining hospitalized at the time of data entry).

### Study design and population

We included all patients hospitalized with laboratory-confirmed or clinically suspected COVID-19 from 2 August 2021, to 3 October 2021 (Delta period), and from 15 November 2021, to 16 February 2022 (Omicron period). These periods corresponded to times when the Delta or Omicron variants accounted for over 90% of circulating cases, as determined by sequencing data from GISAID (https://gisaid.org/hcov-19-variants-dashboard/).

Cases were classified as severe or critical according to a modified definition from the WHO Clinical Management Guidelines for COVID-19. Criteria for severe cases included one or more of the following conditions assessed at hospital admission: SpO_2_ less than 90%; respiratory rate greater than 30 breaths/min for adults and children over 5 years old (≥40 breaths/min in children aged 1–5 years, ≥50 breaths/min in children aged 2 years to 11 months, and ≥60 breaths/min in children <2 months); exposure to extracorporeal membrane oxygenation; ICU admission; use of inotropes or vasopressors; and oxygen therapy (either invasive or noninvasive ventilation). Cases not meeting these criteria were categorized as mild or moderate.

Data collection was conducted using a combination of retrospective and prospective methods, with data entry training provided by WHO to numerous facilities in low-income and middle-income countries (LMICs). The analysis plan was granted a waiver from ethical review by the WHO Ethical Review Committee due to the passive and anonymized nature of the clinical surveillance. Additional ethical approval was obtained from relevant institutional or national review boards where required.

### Statistical analysis

We compared patient characteristics, disease severity, and outcomes of patients admitted during the pre-Delta, Delta, and Omicron period by HIV status. Descriptive statistics and regression analyses were conducted to summarize demographic and clinical characteristics by HIV status and by variant type, and to evaluate their association with the primary outcome – in-hospital mortality. Records with missing data were excluded when determining distributions across outcome levels. Chi-square tests and Student's *t* tests were used to assess the relationship between clinical characteristics and primary outcomes.

The primary objective was to assess whether the risk of in-hospital mortality varied by HIV status across different SARS-CoV-2 variants waves (Omicron, Delta, and pre-Delta). We used flexible parametric survival models (Royston–Parmar models) with natural cubic splines (3 degrees of freedom) to model the baseline hazard, with cluster-robust standard errors to account for clustering by country. In-hospital mortality was defined as death with confirmed or suspected COVID-19 recorded on the death certificate, or death from any cause within 28 days of hospital admission.

Confounders were identified using directed acyclic graphs (DAGs), with age, sex, and severity at admission included as essential adjustment variables. Additional covariates selected based on their data completeness (>80% of cases), low collinearity with other variables (correlation coefficient <0.8), and univariate association (*P* < 0.10) with in-hospital mortality or the primary exposure. Conditions considered for inclusion as covariates included: chronic comorbidities (asplenia, asthma, chronic cardiac, kidney, liver, pulmonary, or neurological disease); lifestyle factors (e.g. smoking); endocrine/metabolic conditions (e.g. diabetes, hypertension, obesity); malignant neoplasms; tuberculosis (current or prior infection). Significant covariates at *P* < 0.05 level were retained in the final model.

Additionally, we conducted regression analyses to assess risk factors for in-hospital mortality among PWH during the Omicron and Delta periods using the same methodological approach. Secondary analyses on the effect of COVID-19 vaccine on reduction of in-hospital mortality and risk of in-hospital mortality by level of CD4^+^ cell count among PWH was assessed using Cox regression analyses. All analyses were performed using SAS version 9.4 (Copyright© 2016 by SAS Institute Inc., Cary, North Carolina, USA) or R version 3.6.3 (Copyright© 2021 by RStudio, Boston, Massachusetts, USA, https://www.R-project.org).

## Results

### Study population

Of 823 845 hospitalized COVID-19 patients included in the global database, a total of 653 272 with known HIV status were included in the analyses. This included 16 361 children and 447 936 adults from the pre-Delta variant wave, 5336 children and 85 122 adults from the Delta variant wave, and 14 551 children and 74 188 adults from the Omicron variant wave.

### HIV co-infection

Overall, 9.1% (*n* = 59 143) of hospitalized patients with COVID-19 were co-infected with HIV, including 17% (6181 of 36 329) of hospitalized children under 18 years. During the Omicron wave, the proportion of PWH was 14.4% (12 733 of 88 739), which was significantly higher compared to both the pre-Delta (8.2%, 38 804 of 472 356; *P* < 0.001) and the Delta variant wave (7.5%, 6800 of 90 458; *P* <0.001). The proportion of PWH was also significantly higher during the pre-Delta wave compared to the Delta variant wave (*P* < 0.001). PWH were more likely to be younger, have a higher comorbidity burden, and be less likely to have been vaccinated across all variant waves (Table 1).

**Table 1:** Characteristics, underlying conditions, and outcomes of people hospitalized with suspected or confirmed COVID-19, by HIV status.

	Pre-Delta waves [*n* (%)]		Delta wave ]*n* (%)]		Omicron wave [*n* (%)]	
Characteristic	HIV +ve	HIV −ve	HIV +ve	HIV −ve	HIV +ve	HIV +ve
Age						
<=18 years	13 866 (3.3)	2495 (6.4)	4289 (5.2)	1047 (15.4)	11 923 (15.8)	2628 (20.7)
>18 to <45 years	94 377 (22.2)	13 034 (33.6)	20 782 (25.1)	2282 (33.6)	21 812 (29.0)	4170 (32.8)
>45 to <65 years	151 758 (35.7)	15 171 (39.2)	29 049 (35.1)	2058 (30.3)	17 275 (22.9)	2745 (21.6)
>65 to <75 years	71 407 16.8)	4638 (12.0)	13 601 (16.5)	682 (10.0)	9978 (13.2)	1320 (10.4)
>75 years	94 142 (22.1)	3409 (8.8)	14 937 (18.1)	726 (10.7)	14 361 (19.1)	1861 (14.6)
Gender						
Female	209 929 (48.5)	19 612 (50.6)	42 934 (51.4)	3784 (55.7)	41 367 (54.5)	6705 (52.7)
Male	223 245 (51.5)	19 153 (49.4)	40 654 (48.6)	3009 (44.3)	34 595 (45.5)	6021 (47.3)
Disease severity						
Mild/moderate	238 818 (55.1)	9281 (23.9)	50 274 (60.1)	2128 (31.3)	59 885 (78.8)	4031 (31.7)
Severe/critical	194 734 (44.9)	29 523 (76.1)	33 384 (39.9)	4672 (68.7)	16 121 (21.2)	8702 (68.4)
Chronic cardiac disease						
No	62 408 (84.6)	8433 (75.5)	22 644 (96.8)	9126 (77.7)	21 516 (97.3)	12 613 (89.5)
Yes	11 363 (15.4)	2732 (24.5)	760 (3.3)	2626 (22.4)	591 (2.7)	1478 (10.5)
Diabetes mellitus						
No	57 537 (78.1)	8116 (72.2)	18 821 (84.4)	8196 (69.5)	19 425 (90.5)	11 458 (80.2)
Yes	16 151 (21.9)	3123 (27.8)	3487 (15.6)	3598 (30.5)	2029 (9.5)	2826 (19.8)
Hypertension						
No	46 933 (63.9)	5905 (52.7)	16 643 (74)	6079 (51.8)	17 789 (82)	9210 (63.3)
Yes	26 561 (36.1)	5300 (47.3)	5859 (26)	5663 (48.2)	3895 (18)	5350 (36.7)
Chronic pulmonary disease						
No	65 939 (91.0)	9650 (86.8)	21 569 (97.6)	9956 (84.3)	21 066 (97.7)	13 558 (92.8)
Yes	6518 (9.0)	1468 (13.2)	523 (2.4)	1855 (15.7)	501 (2.3)	1057 (7.2)
Current and latent TB						
No	33 523 (97.8)	3621 (99.4)	16 446 (97.5)	3230 (98.7)	19 136 (96.4)	10 924 (98.1)
Yes	755 (2.2)	23 (0.6)	424 (2.5)	42 (1.3)	716 (3.6)	214 (1.9)
Asthma						
No	66 581 (91.2)	9620 (88.4)	2855 (67.3)	10 029 (85.8)	20 879 (96.4)	13 300 (92.6)
Yes	6457 (8.8)	1265 (11.6)	1390 (32.7)	1661 (14.2)	788 (3.6)	1069 (7.4)
Chronic kidney disease						
No	66 020 (90.9)	9447 (84.4)	21 599 (97.8)	9995 (85.2)	21 146 (98.0)	13 591 (92.9)
Yes	6610 (9.1)	1752 (15.6)	478 (2.2)	1740 (14.8)	422 (2.0)	1043 (7.1)
Chronic liver disease						
No	46 122 (97.1)	9192 (97.2)	6016 (97.7)	8761 (96.5)	3928 (98.8)	5734 (96.9)
Yes	1371 (2.9)	264 (2.8)	142 (2.3)	315 (3.5)	47 (1.2)	182 (3.1)
Chronic neurological disorder						
No	42 535 (91.0)	8321 (89.4)	5822 (95.0)	8132 (90.3)	3859 (95.8)	5510 (91.3)
Yes	4184 (9.0)	982 10.6)	305 (5.0)	870 (9.7)	170 (4.2)	526 (8.7)
Malignant neoplasm						
No	68 917 (93.1)	9689 (89.0)	22 790 (98.9)	10 657 (90.7)	21 941 (96.6)	13 839 (94.8)
Yes	5101 (6.9)	1194 (11.0)	250 (1.1)	1093 (9.3)	765 (3.4)	756 (5.2)
COVID-19 vaccine						
No	71 541 (86.8)	3393 (87.5)	20 848 (64.3)	2252 (88.9)	20 104 (58.9)	2673 (71.4)
Yes	10 859 (13.2)	483 (12.5)	11 596 (35.7)	282 (11.1)	14 048 (41.1)	1071 (28.6)
Mortality						
Discharged	342 629 (79.0)	29 426 (75.8)	67 831 (81.1)	5304 (78.0)	69 966 (92.1)	10 272 (80.7)
Deceased	90 923 (21.0)	9378 (24.2)	15 827 (18.9)	1496 (22.0)	6040 (8.0)	2461 (18.3)

44.1% of hospitalized patients had severe or critical COVID-19 disease. The proportion of PWH hospitalized with severe or critical disease was significantly higher at 73.8% (43 655 of 59 143) compared to 41.2% (244 584 of 594 129; *P* *<* 0.001) in the HIV-negative population across all variant waves (Table 1).

### Children's in-hospital mortality across variant waves

**Fig. 1:**
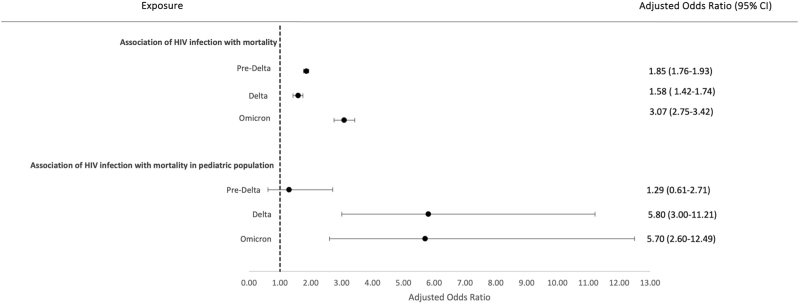
Association of HIV status with in-hospital mortality across pre-Delta, Delta, and Omicron variant periods.

Overall, 1.6% (581/36 329) of children hospitalized with COVID-19 died during their hospital stay being 1.93% (315/16 361) during the pre-Delta waves, 1.82% (97/5336) during the Delta wave and 1.04% (151/14 551) during the Omicron wave. Mortality rate for children with HIV (CWH) was higher compared to the HIV-negative children across all variant waves (2.7 vs. 1.8% during the pre-Delta waves, 2.3 vs 1.7% during the Delta wave, and 1.6 vs. 0.9% during the Omicron wave). Overall, the risk of in-hospital mortality was 480% higher during Delta (aHR 5.80, 95% CI 3.0–11.21), 470% higher (aHR 5.70, 95% CI 2.60–12.49) during the Omicron variant wave, and 29% higher during pre-Delta waves (aHR 1.29, 95% CI 0.61–2.71) among CWH compared to HIV-negative children. The risk of in-hospital death during the pre-Delta waves was, however, nonsignificant (Fig. 1).

### In-hospital mortality across variant waves

PWH exhibited a higher risk of in-hospital mortality compared to HIV-negative patients across all variant waves. Overall, 19.4% (126 641/653 272) of those hospitalized with COVID-19 died during their hospital stay. The proportion of in-hospital deaths was 21.2% (100 301/472 356) during pre-Delta waves, 19.3% (17 323/90 008) during the Delta wave, and 9.6% (8501/88 379) during the Omicron wave. Mortality rate was higher among PWH compared to the HIV-negative across all variant waves (24.2 vs. 21% during the pre-Delta waves, 22 vs. 18.9% during the Delta wave, and 19.3 vs. 8% during the Omicron wave). The risk of in-hospital mortality was 85% higher during the pre-Delta waves (aHR 1.85, 95% CI 1.76–1.93), 58% higher (aHR 1.58, 95%CI 1.42–1.74) during the Delta wave, and 207% during the Omicron variant wave (aHR 3.07, 95% CI 2.75–3.42) compared to HIV-negative individuals. The risk of in-hospital death during the pre-Delta waves was, however, nonsignificant (Fig. 1).

Among HIV-negative patients, in-hospital deaths decreased by 10% during Delta variant wave compared to the pre-Delta waves and by 58 and 62% during the Omicron wave, relative to the pre-Delta and Delta waves, respectively. In contrast, the reduction in in-hospital mortality among PWH was more modest: a decrease of 9% during the Delta wave compared to the pre-Delta wave, and a 12 and 20% reduction during the Omicron wave relative to the pre-Delta and Delta waves.

Notably, the risk of in-hospital death among PWH was particularly higher among those with low CD4^+^ cell counts 200 cells/mm^3^ or less compared with those with CD4^+^ cell counts of at least 200 cells/mm^3^ relative to the HIV-negative patients (aHR 2.38, 95% CI 2.16–2.61 vs. aHR 1.29, 95% CI 1.18–1.41 during pre-Delta waves, aHR 1.85, 95% CI 1.49–2.31 vs. aHR 1.31, 95% CI 1.07–1.59 during Delta and aHR 3.97, 95% CI 3.34–4.73 vs. aHR 2.51, 95% CI 1.98–3.19 during the Omicron variant wave, respectively. However, the percentage reduction in the mortality risk across the different SARS-CoV-2 variant waves did not differ by CD4^+^ cell count levels (proportion of in-hospital mortality; 22, 20, and 14% for those with CD4^+^ cell count greater than 200 cells/mm^3^ vs. 34, 31, and 25% for those with CD4^+^ cell counts 200 cells/mm^3^ or less across pre-Delta, Delta, and Omicron waves. respectively.

### Risk factors associated with in-hospital death across variant waves

Table 2 shows risk factors associated with in-hospital death among PWH across all the three variant waves. Common risk factors across pre-Delta, Delta and Omicron waves were male gender, increasing age (18–45, 45–65, 65–75, and over 75 years relative to those under 18 years), having severe or critical COVID-19 illness at hospital admission and chronic kidney disease. Co-infection with active or latent TB was a common risk factor during the pre-Delta and Omicron waves, while diabetes was a common risk factor during the pre-Delta and Delta waves.

**Table 2:** Risk factors for in-hospital deaths among people with HIV hospitalized with COVID-19 disease during the pre-Delta, Delta, and Omicron SARS-CoV-2 variant waves.

	Pre-Delta					Delta				Omicron		
Characteristic	*N*	Deaths [*n* (%)]	aHR (95% CI)	*P* value	*N*	Deaths [*n* (%)]	aHR (95% CI)	*P* value	*N*	Number of deaths	aHR (95% CI)	*P* value
Sex												
Female	19 612	4494 (23)	1.0		3785	809 (21)	1.0		6705	1207 (18)	1.0	
Male	19 153	4879 (25)	**1.15 (1.08–1.21)**	**<0.001**	3009	686 (23)	**1.24 (1.06–1.44)**	**<0.001**	6021	1252 (21)	**1.21 (1.10–1.32)**	**<0.001**
Age												
≤18 years	2495	68 (2.7)	1.0		1046	24 (2.3)	1.0		2628	42 (1.6)	1.0	
>18–45 years	13 032	2118 (16)	**6.74 (4.88–9.31)**	**<0.001**	2282	426 (19)	**6.30 (3.82–10.40)**	**<0.001**	4163	683 (16)	**11.8.0–16.89)**	**<0.001**
>45–65 years	15 150	3956 (26)	**10.77 (7.81–14.86)**	**<0.001**	2055	576 (28)	**9.86 (6.03–16.12)**	**<0.001**	2745	658 (24)	**17.04 (11.82–24.56)**	**<0.001**
>65–75 years	4635	1747 (38)	**17.17 (12.42–23.75)**	**<0.001**	681	220 (32)	**14.71 (8.97–24.13)**	**<0.001**	1320	372 (28)	**25.15 (17.49–36.16)**	**<0.001**
>75 years	3408	1481 (43)	**21.85 15.80–30.22)**	**<0.001**	726	249 (34)	**20.75 (12.79–33.66)**	**<0.001**	1861	703 (38)	**35.32 (24.77–50.38)**	**<0.001**
Severity at admission												
Mild/moderate	9261	1444 (16)	1.0		2125	355 (17)	1.0		4029	568 (14)	1.0	
Severe/critical	29 516	7932 (27)	**2.10 (1.90–2.44)**	**<0.001**	4670	1140 (24)	**2.02 (1.68–2.41)**	**<0.001**	8697	1891 (22)	**2.28 (1.93–2.70)**	**<0.001**
TB												
No	26 291	5930 (2.3)	1.0		3938	763 (19)			8727	1612 (18)	1.0	
Yes	1946	572 (29)	**1.36 (1.22–1.52)**	**<0.001**	497	138 (28)			1142	282 (25)	**1.57 (1.31–1.88)**	**<0.001**
Diabetes												
No	26 536	5757 (22)	1.0		4148	759 (18)	1.0		8675	1536 (18)		
Yes	7991	2591 (32)	**1.15 (1.09–1.21)**	**<0.001**	1040	325 (31)	**1.24 (1.09–1.41)**	**0.006**	1848	510 (28)		
Chronic kidney disease												
No	32 349	7413 (23)	1.0		4785	919 (19)	1.0		9764	1838 (19)	1.0	
Yes	1137	482 (58)	**1.52 (1.35–1.70)**	**<0.001**	188	78 (41)	**1.66 (1.28–2.15)**	**<0.001**	367	123 (34)	**1.30 (1.06–1.58)**	**0.010**

aHR, adjusted hazard ratio. bold; significant risk factors for in-hospital COVID-19 among PWH

### Association of COVID-19 vaccination and in-hospital mortality

Data on COVID-19 vaccination status was available for 193 980 hospitalized patients, with 11% (11 761 of 103 382) vaccinated during the pre-Delta wave, 28.2% (12 614 of 44 787) during the Delta wave, and 28% (12 821 of 45 811) during the Omicron variant wave. COVID-19 vaccination was associated with a significant reduction in the risk of death: 40% reduction (aHR 0.60, 95% CI 0.57–0.64) during the pre-Delta wave, 38% reduction (aHR 0.62, 95% CI 0.57–0.67) during the Delta wave, and 42% reduction (aHR 0.58, 95% CI 0.53–0.64) during the Omicron wave. Among PWH, a similar pattern was observed, with a 60% reduction (aHR 0.40, 95% CI 0.31–0.51) in the risk of death during the pre-Delta wave, 50% reduction (aHR 0.50, 95% CI 0.33–0.75) during the Delta wave, and 54% reduction (aHR 0.46, 95% CI 0.36–0.59) during the Omicron variant wave.

## Discussion

Our findings show a persistently higher risk of in-hospital mortality among PWH hospitalized with COVID-19 compared to HIV-negative individuals across all SARS-CoV-2 variant waves. While overall mortality declined during the Omicron wave compared to the pre-Delta and Delta waves, the reduction was modest among PWH. In contrast, the decrease in risk of death during the Delta variant wave, relative to the pre-Delta wave, was modest and similar for both PWH and HIV-negative populations.

Previous studies have reported conflicting findings regarding the increased risk of severe and fatal COVID-19 outcomes among PWH [9,11–13]. However, our study consistently shows a high mortality risk across all variant waves, aligning with a recent large systematic review [9], which included over 20 million COVID-19 patients from Africa, Asia, Europe, and North America and found a 78% increased mortality risk and a higher risk of hospitalization for COVID-19 among PWH. Notably, our study found that PWH had a consistent high risk of mortality regardless of CD4^+^ cell count, although those with lower CD4^+^ cell counts (<200 cells/mm^3^) faced a greater mortality risk. These findings support existing evidence prioritizing PWH for COVID-19 vaccines and therapeutics [20].

The highest in-hospital mortality rates occurred during the pre-Delta wave for both HIV-negative and PWH, suggesting that the initial variant waves may have been more severe than the Delta wave, as reported in other studies [21–23]. The reduction in in-hospital mortality during the Delta waves may reflect some protective immunity acquired from past infections, increased vaccine coverage, or improved disease management and treatment over time. Our findings align with other studies showing significant declines in disease severity and mortality during the Omicron period [24–29].

During the Omicron wave, we observed an increase in the number of hospitalized cases among children, particularly those under 15 years of age, which may reflect the high transmissibility of the Omicron variant and the lower vaccination coverage in this age group [30,31]. In many countries, children were not prioritized for vaccination due their relative better prognosis compared to adults, and in some cases, were not eligible pending safety data from clinical studies. Despite the increased number of hospitalized children, the overall proportion of in-hospital deaths declined during the Omicron period. Factors contributing to this decline may include immunity from past infections, relative higher vaccination coverage, and improved clinical management, alongside the lower intrinsic virulence of the Omicron variant[32–36].

Despite a general decline in mortality during the Omicron wave, the reduction was only modest among both children and adults living with HIV, and the prevalence of HIV hospitalized cases was also significantly higher during the Omicron wave. This suggests potential differences in pathogenicity for the Omicron variant in PWH compared to the HIV-negative populations or that the Omicron variant, speculated to have emerged from an HIV-infected individual, may have adapted better to this demographic. Immune responses in PWH, whether from past infections or vaccines, tend to be lower than those in HIV-negative individuals due to compromised immune systems affecting antibody and T-cell production [37–39].

Mortality risk remained high among hospitalized PWH with severe COVID-19, male gender, older age, and those with underlying comorbidities, particularly chronic kidney disease, across all variant waves. While there was a notable decline in mortality rates among these high-risk groups during the Omicron wave [37,38], our findings underscore the importance of prioritizing them for vaccination, including booster doses, during new transmission waves in the postpandemic phase [40,41]. In our study, vaccinated PWH had a 50–60% reduced risk of death across all SARS-CoV-2 variant waves, further re-inforcing the protective benefit of vaccination, as also reported from other studies [42,43].

There are some limitations to our study. Firstly, use of variant dominance period as a proxy for variant exposure may affect the precision of the impact of variants on in-hospital mortality due to potential misclassifications. Additionally, our inability to distinguish the dominant variants during the pre-Delta waves mask the specific impact of each variant on the outcomes. Secondly, our study only focused on in-hospital mortality and may not capture the broader community-level effects of SARS-CoV-2 variants on mortality by HIV status, as our sample likely consisted of cases who required and had access to care. Moreover, we were not able to also assess for postdischarge mortality. Due to this, we are not able to draw conclusions of the differential effect of SARS-CoV-2 variants on mortality across the different at-risk populations at the community level. Third, some countries contributed data derived from national registries, while others from a convenience sample of hospitals or sentinel clinics. This coupled with differences in hospitalization criteria and access and quality of healthcare by country may result to potential reporting biases. Moreover, the differences in care across countries may potentially limit the generalizability of these findings to other contexts rather than the countries included in the study. Fourth our dataset had missing information on antiretroviral therapy (ART), CD4^+^ cell counts, viral load and prior COVID-infection minimizing the ability to comprehensively assess the effect of these risk factors, which have been associated with mortality risk among PWH in some studies [44–48]. Finally, this study only assessed mortality during hospitalization. Studies have shown increased risk of mortality among PWH during the early postdischarge period, suggesting a conservative mortality estimate from our study [49].

In conclusion, our study highlights a consistently high in-hospital mortality risk among PWH compared to the HIV-negative individuals during all the SARS-CoV2 variant waves. While the risk of death among HIV-negative people decreased drastically in the Omicron wave, the reduction in in-hospital mortality among PWH was only modest. Overall, this emphasizes the ongoing need to prioritize PWH for COVID-19 vaccines and medical interventions, especially those with low CD4^+^ cell counts and severe disease. Our findings suggest that tailored public health strategies and targeted vaccination campaigns are essential to protect this at-risk group effectively. Continued surveillance and research are necessary to deepen our understanding of the evolving impact of SARS-CoV-2 variants on different populations, including the potential differences in immune responses among PWH. Addressing these challenges will be critical in mitigating the effects of future waves of COVID-19 and ensuring equitable health outcomes for all individuals, particularly those facing compounded vulnerabilities due to HIV status.

## Acknowledgements

We express our sincere appreciation to all Member States, facilities, and collaborators who contributed individualized, anonymized data of hospitalized patients with suspected or confirmed COVID-19 to the WHO Global Clinical Platform for COVID-19. The full list of facilities that contributed to this analysis is provided on the Platform webpage[50]. We are also grateful to Flaminia Sabbatucci and Firdavs Kurbonov for their contribution to support the WHO Platform outreach, communication, data cleaning, and verification. Finally, we are grateful to the members of the Clinical Advisory Group for their clinical feedback on all aspects of the WHO Platform and Chiori Kodama for their facilitation of data contribution at the WHO regional levels.

Contributors: S.B., J.D., S.I., R.S., and S.S.T. conceptualized the study, R.S. conducted the analyses, S.I. drafted the manuscript with inputs from all the co-authors (R.S., N.F., S.S.T., J.W., A.Z., J.D., M.D., and S.B.). All authors had final responsibility for the decision to submit for publication. All authors reviewed and contributed to subsequent drafts for important intellectual content and approved the final manuscript.

The views presented in this Article are those of the authors and do not reflect those of the WHO or the Pan American Health Organization.

Role of the funding source: No funding was sought or obtained for this study.

### Conflicts of interest

There are no conflicts of interest.
